# A 3D Collagen-Based In Vitro Cancer Model Created Through Modular Tissue Engineering

**DOI:** 10.3390/cancers18060935

**Published:** 2026-03-13

**Authors:** Nima Daneshvar Baghbadorani, Mira Bosso, Rowen Greene, Taylor Dzikowski, Breanne Bevelander, Amelia Gagnon, Morgan Johannson, Mohammadreza Javan, Parnaz Soori, Michael Dean Chamberlain

**Affiliations:** 1 Cancer Research Group, University of Saskatchewan, 107 Wiggins Road, Saskatoon, SK S7N 5E5, Canada; tac912@mail.usask.ca (N.D.B.); mira.bisso@usask.ca (M.B.); rrg769@mail.usask.ca (R.G.); uyc271@mail.usask.ca (T.D.); breanne.bevelander@usask.ca (B.B.); akgagnon@ualberta.ca (A.G.); djx547@usask.ca (M.J.); ltm268@mail.usask.ca (M.J.); dov230@usask.ca (P.S.); 2 Department of Health Sciences, College of Medicine, University of Saskatchewan, 107 Wiggins Road, Saskatoon, SK S7N 5E5, Canada; 3 Department of Biochemistry, Microbiology & Immunology, College of Medicine, University of Saskatchewan, 107 Wiggins Road, Saskatoon, SK S7N 5E5, Canada; 4 Department of Anatomy, Physiology and Pharmacology, College of Medicine, University of Saskatchewan, 107 Wiggins Road, Saskatoon, SK S7N 5E5, Canada; 5 Department of Oncology, College of Medicine, University of Saskatchewan, 107 Wiggins Road, Saskatoon, SK S7N 5E5, Canada; 6 Department of Discovery and Translational Research, Saskatchewan Cancer Agency, 107 Wiggins Road, Saskatoon, SK S7N 5E5, Canada

**Keywords:** 3D cell culture, drug resistance, hypoxia, microtissues, tumour microenvironment (TME), collagen, cancer stem cells (CSCs)

## Abstract

Background: An emerging tool to better simulate the complexity of tumour biology in vitro is 3D culture models. Several approaches have been introduced, yet many face challenges such as technical complexity or limited ability to reproduce critical tumour traits. Modular tissue engineering is a well-known method in tissue transplantation, where it has been used to develop various healthy tissue constructs. In this study, we set out to adapt this established approach to fabricate cancer microtissues and to assess their effectiveness as a tumour model that can capture essential features of cancer biology and drug-treatment response. Methods: Two triple-negative breast cancer (TNBC) cell lines, HCC1806 and MDA-MB-231, were cultured in microtissues and assessed for viability, cell death, generation of hypoxia and response to chemotherapy. To benchmark our model, we utilized flow cytometry to analyze the CD44^+^/CD24^−^ cancer stem cell (CSC) phenotype across microtissues, 2D monolayers, and established 3D models, including spheroids, collagen domes, and laminin-rich domes. Results: The cells showed sustained cell viability with minimal cell death, along with natural development of tumour properties, such as hypoxia. Crucially, flow cytometry revealed a cell-line-dependent regulation of the CD44^+^/CD24^−^ phenotype, underscoring the complex influence of the 3D microenvironment on stem cell regulation. Furthermore, by screening the model with standard anti-breast cancer chemotherapeutics, we observed drug resistance at concentrations comparable to those used in the clinic. Conclusions: Our model offers the unique ability to spontaneously reproduce fundamental features of tumours in vitro, capturing the cellular heterogeneity and reprogramming that drive clinical drug resistance.

## Simple Summary

Most new cancer drugs fail in clinical trials, largely because preliminary drug studies do not adequately reflect the complexity of this human pathology. In this context, three-dimensional culture systems have advanced cancer research, but many existing models are difficult to fabricate or fail to reproduce important tumour characteristics. In this study, we developed cancer microtissues, which are small, free-floating collagen hydrogels containing cancer cells. They are produced through an efficient process known as modular tissue engineering and naturally replicate key features of real tumours, including low oxygen regions, stemness phenotypes and resistance to treatment. Our findings highlight the potential of microtissues as a practical model that can be used as an alternative to other three-dimensional cancer culture systems.

## 1. Introduction

The majority of novel drug candidates, especially anti-cancer therapies, fail to establish clinical therapeutic efficacy [1,2]. This issue is partly due to the inaccurate preliminary assessment of novel drug candidates by techniques and models that are not able to resemble the complexity of human pathologies [3,4]. Despite the historical success of 2D-cultured cancer cell lines in advancing our understanding of cancer biology, they have inherent drawbacks [5]. The major limitation of 2D cell culture is its inability to account for the heterogeneity of tumours. An actual tumour is a heterogeneous population of various cell types, including cancer, stromal and immune cells, supported by a three-dimensional (3D) network of macromolecules, the extracellular matrix (ECM) [5,6,7]. The 3D geometry of the tumour allows interaction between cells and their surrounding microenvironment, and the establishment of nutrient and oxygen gradients [8]. Additionally, this 3D microenvironment is a major determinant of tumour cell behaviour and promotes cancer progression, stemness, therapy resistance, and disease relapse [9,10,11,12,13]. In contrast, 2D cancer models consist of a homogeneous monolayer of adherent cancer cells in a dish with unlimited access to oxygen and nutrients, and in the absence of cancer-associated stromal cells and ECM that make up the tumour microenvironment (TME) [5]. Although in vivo studies are typically used to account for this lost complexity in preliminary drug studies, the use of in vivo models imposes ethical, time, and financial constraints that limit their utility [14]. Moreover, they provide us with a partly functional TME which is similar to the human one, but not identical [15,16]. Accordingly, the development of 3D in vitro models able to mimic the main features of TME, including the ECM, oxygen and nutrient gradients, hypoxia, and immune cell surveillance, has been considered as a solution to overcome limitations of 2D in vitro and in vivo models.

Recent advances in tissue-engineered 3D models enhance the precise reproduction of tumour cell behaviour in vitro by providing a more physiologically relevant and well-characterized TME. These models have different levels of complexity, ranging from simple tumour spheroids [17] to more advanced scaffold-based models [18,19], microfluidics [20,21,22], and organoids [23,24,25]. Among the variety of introduced scaffolds for 3D cell culture, collagen-based hydrogels have been shown by several studies [26,27,28,29] to serve as promising biocompatible scaffolds capable of recapitulating key aspects of the solid tumour microenvironment. However, accurately reconstructing the specialized tumour niches required to maintain dynamic cancer stem cell (CSC) phenotypes remains a significant challenge in many of these systems [30,31]. CSCs represent a subpopulation of tumour cells with self-renewal capacity, tumour-initiating potential, and increased resistance to therapy, making their preservation in vitro critical for modelling tumour biology and drug response. Collagen is the most abundant mammalian ECM protein, playing important roles in cell signalling, migration, and adhesion [32,33,34]. Furthermore, collagen deposition is upregulated in the TME of several tumour types [35].

Our study is focused on implementing modular tissue engineering designs in cancer research to overcome some of the limitations of conventional 3D cell culture methods. The model system stemming from this approach is called microtissues, a free-floating cylindrical collagen hydrogel that can be embedded with cancer cells and other cell types to mimic the TME. Similar to 2D cell culture, the microtissues are easy to use and manipulate, can be used in any standard molecular or cellular assay, and are cost-effective with no need for expensive manufacturing equipment. Modular tissue engineering has previously demonstrated translational utility across multiple tissue systems. For example, sub-millimetre collagen modules populated with endothelial cells have been successfully assembled into larger vascularized constructs capable of supporting perfusion and integration following implantation [36]. These modular systems enabled rapid extracellular matrix remodelling, sustained cell viability, and scalable fabrication, highlighting their potential as reproducible building blocks for complex tissue modelling. Such features make modular platforms particularly attractive for tumour modelling, where controlled architecture, diffusion gradients, and cell–matrix interactions are critical determinants of phenotype and drug response. Although this technique was initially developed for tissue engineering and transplantation research [36,37,38,39], it offers several advantages for 3D tumour cell culture by addressing key limitations of commonly used 3D platforms in cancer research (Table 1).

As shown in Table 1, conventional 3D culture systems frequently present practical limitations that affect reproducibility and experimental consistency. Spheroid systems, while simple to implement, often exhibit aggregation-dependent size variability that can influence downstream analyses. Laminin-rich matrices such as Matrigel (or Geltrex) exhibit batch-to-batch variability, as their growth factor composition can differ between batches [40]. This inherent variability may affect experimental reproducibility. In contrast, collagen-based systems provide a more defined extracellular matrix environment with reduced compositional variation. Also, collagen is the most abundant ECM within breast cancer tumours [41,42]. Within this framework, modular tissue engineering enables a higher fabrication yield per millilitre of collagen while ensuring consistent construct geometry across batches through the use of a standardized cutting plate. Furthermore, the free-floating configuration of microtissues permits cell-mediated contraction and matrix remodelling, thereby enhancing the physiological relevance of the model.

In this paper, we describe for the first time the features of the initial version of microtissues used for tumour modelling. This basic model contains only cancer cells embedded in the collagen hydrogel. Using this model, we cultured the triple-negative breast cancer (TNBC) cell lines, HCC1806 and MDA-MB-231, which represent biologically divergent models of triple-negative breast cancer. HCC1806 exhibits a more epithelial-like phenotype, whereas MDA-MB-231 displays a mesenchymal-like, highly invasive profile, thereby enabling assessment of the platform across phenotypically distinct tumour states. We assessed microtissues for viability and cell death, morphological alterations, stemness, emergence of hypoxia, and chemoresistance.

## 2. Materials and Methods

### 2.1. Drugs and Reagents

Stock solutions of vincristine (Focus Biomolecules, Plymouth Meeting, PA, USA, 10-2570) and vinblastine (LC Laboratories, Woburn, MA, USA, V-7300) were prepared in sterile water. Stock solutions of paclitaxel (Cayman Chemical, Ann Arbor, MI, USA, 10461) and doxorubicin (APExBIO, Houston, TX, USA, A3966) were prepared in DMSO (Sigma-Aldrich, St. Louis, MO, USA). All stock solutions were kept at −80 °C.

### 2.2. 2D Cell Culture

Experiments were conducted using two TNBC cell lines, and both were cultured using the specifications outlined by the American Type Culture Collection (ATCC). The HCC1806 cell line was cultured in RPMI 1640 (Gibco, Grand Island, NY, USA, SH30027.01) medium supplemented with 10% fetal bovine serum (FBS; Gibco, 12483-020) and 1% Penicillin/Streptomycin (Invitrogen, Waltham, MA, USA, SV30010) and was maintained in a humidified atmosphere containing 5% CO_2_ at 37 °C. The MDA-MB-231 cell line was cultured in L-15 Leibovitz (Gibco, 11415-064) medium supplemented with 10% FBS and 1% Penicillin/Streptomycin and was maintained in a non-CO_2_ incubator at 37 °C. All cell lines were obtained from ATCC (Manassas, VA, USA).

### 2.3. 3D Cell Culture

#### 2.3.1. Microtissues

**Figure 1:**
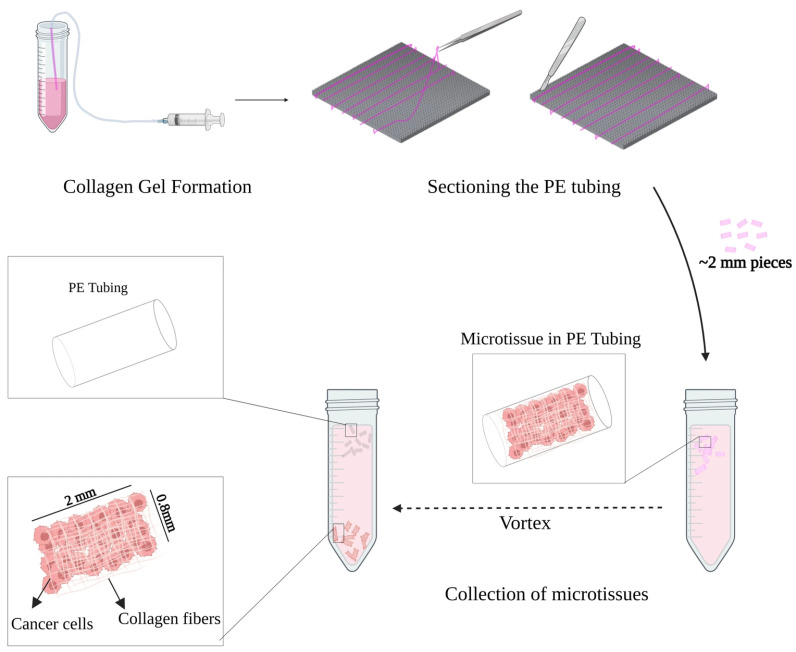
Schematic representation of the modular tissue engineering 3D-printed cutting plate. Once the collagen gel is formed, the tubing is placed into the cutting plate and cut into 2 mm pieces. The mixture of collagen and cancer cells is infused into a sterile polyethylene tubing. Once the collagen is gelled, the tubing is sectioned into 2 mm pieces, collected in media, and vortexed to release the microtissues from the tubing and created with BioRender.com.

Microtissues of HCC1806 or MDA-MB-231 were fabricated similarly to previous methods [43,44] with a few modifications. Briefly, Type I bovine Collagen (3 mg/mL, PureCol^®^, Advanced BioMatrix, San Diego, CA, USA, 5005) solution was mixed with 10X minimum essential media (MEM; Gibco, M9288) stock solution to a 1× MEM solution and neutralized by 1 M sodium bicarbonate solution to achieve pH 7.4 and kept on ice to prevent gelation. The cells were added at a density of 2 million cells/mL of collagen, the mixture was loaded into sterile polyethylene tubing (PE90, BD Biosciences, Franklin Lakes, NJ, USA, 14117012D) and incubated in a humidified atmosphere at 37 °C and 5% CO_2_ for 1 h to gel. The tubing was then cut using a specialized 3D-printed cutting plate (Figure 1). The cutting plate holds the tubing and has cutting tracks that guide the razor blade through designated slots, which were 2 mm apart. The cut pieces were collected in media in a 50 mL tube and vortexed to remove the tubing from the microtissues. The microtissues were then maintained in the conditions required by the cell line as outlined by the ATCC.

#### 2.3.2. Hydrogel Domes

For collagen domes, the same Collagen mixture prepared for the fabrication of microtissues was used, and for laminin-rich domes, Geltrex LDEV-Free Reduced Growth Factor Basement Membrane Matrix (Gibco) was utilized. In both conditions, cells were resuspended in the unpolymerized hydrogel at a density of 2 million cells/mL. Droplets (20 µL) were manually pipetted onto the centre of culture wells. Immediately following dispensing, the culture plates were inverted and incubated at 37 °C for 20 min. This inversion step was performed to counteract gravity-induced cell sedimentation during the gelation phase, ensuring uniform cellular distribution throughout the 3D matrix. Once polymerization was complete, plates were returned to the upright position, and the proper culture medium was added.

#### 2.3.3. Spheroids

Spheroids were generated using ultra-low attachment (ULA) 96-well plates (Corning, Corning, NY, USA). TNBC cells were seeded at a density of 3000 cells in 100 uL of culture medium per well. Following 5 days of incubation under standard culture conditions, well-formed multicellular spheroids were obtained and used for downstream experiments.

### 2.4. Histology

Microtissues were washed with phosphate-buffered saline (PBS) and fixed in 10% neutral buffered formalin (NBF) overnight. The microtissues were then embedded in 1.5% agarose using a cryo-mould. The agarose blocks were fixed in 10% NBF for 24 h, washed in 70% ethanol twice, and left in 70% ethanol until further processing. After processing, slides were either stained for Hematoxylin and Eosin (H&E) or immunohistochemistry (IHC). For IHC, heat-induced epitope retrieval was performed by microwave treatment in sodium citrate buffer (10 mM sodium citrate, 0.05% Tween 20, pH 6.0), followed by washing and blocking of endogenous peroxidases with 3% hydrogen peroxide. Tissue sections were then blocked with 3% bovine serum albumin (BSA) in PBS and incubated with active Caspase-3 antibody (1:200, Thermofisher, Waltham, MA, USA, MA5-32015). After washing, slides were incubated with a Horseradish peroxidase (HRP)-labelled secondary antibody (Agilent Technologies, Santa Clara, CA, USA, K400311-2), washed again, and developed with DAB chromogen (Agilent Technologies, K346811-2). Slides were scanned using the Aperio virtual microscope (CS Scanscope, Leica Biosystems, Vista, CA, USA).

### 2.5. RNA-Sequencing

Using the RNeasy Fibrous Tissue Mini Kit (Qiagen, Hilden, Germany), total RNA was harvested from Microtissues at days 5 and 9 and sequenced on an Illumina NovaSeq 6000 platform(Illumina, San Diego, CA, USA) following standard library preparation. Read alignment to the GRCh38 genome was performed using STAR v2.7.10a. Statistical analysis of differential gene expression was conducted using DESeq2 (cutoff: adjusted *p* < 0.05). Two biological replicates of the microtissues were sequenced. The sequences for this project are available from SRA data: PRJNA1414100.

### 2.6. Viability Assay

The viability of microtissues was assessed at day 1, 3, 5, 7, 10, 14 and 21. On each day, three microtissues were transferred to a well of a non-tissue culture-treated 96-well plate in 100 μL of media. At least three wells were considered for each experiment. After seeding, the viability was assessed by the CellTiter-Glo^®^ 3D kit (Promega, Madison, WI, USA) according to its manufacturer’s protocol. Each well received 100 μL of reagent followed by vigorous shaking for 5 min, then microtissues were transferred to an opaque 96-well plate and incubated at room temperature for 25 min. Luminescence was read by the Varioskan LUX multimode microplate reader (Thermofisher).

### 2.7. Live/Dead Staining Assay

Microtissues were transferred to a chambered coverglass-bottomed slide (Lab-Tek™, Nunc, Rochester, NY, USA), washed with PBS, and incubated with Calcein acetoxymethyl ester (1:2000) and SYTOX™ deep red nucleic acid stain (1:2000) for 30 min according to the live/dead kit instructions (Thermofisher, L32250). The assay was performed at days 3, 5, 10, 14 and 21. Following incubation, microtissues were imaged by confocal microscopy (Zeiss LSM700, Carl Zeiss Microscopy GmbH, Jena, Germany).

### 2.8. Flow Cytometry

Microtissues were digested at 5, 14 and 21 days by a stepwise digestion starting with 100U of collagenase type I (Thermofisher, 17018029) in HBSS buffer (Gibco, 14025092), followed by the addition of TrypLE (Gibco, 126404-013) to characterize a single cell suspension. 2D-cultured cells were detached using TrypLE to avoid any effect on cell surface markers. The single-cell suspension was washed in PBS and incubated with Fixable Live/Dead viability dye (Thermofisher, L34955) at 1:1000 in PBS per million cells on ice for 30 min. Next, the cell suspension was washed with FACS buffer (5% FBS in PBS) and was incubated with primary conjugated antibody for CD24 (APC/Cy7, abcam, Cambridge, UK, ab197137), CD44 (PerCP, abcam, ab275662) at a dilution of 5 µL antibody per 100 µL FACS buffer per 1 × 10^6^ cells for 30 min at room temperature. Following incubation, cells were washed and resuspended in FACS buffers. Data acquisition was performed on Beckman CytoFLEX Flow Cytometer (Beckman Coulter, Brea, CA, USA).

### 2.9. Drug Screening Assay

Microtissues were cultured for 5 days, and then three microtissues in 100 μL of culture media were transferred into the wells of a non-tissue culture-treated 96-well plate. In the case of 2D-cultured cells, 1200 cells were seeded per well of a tissue-culture-treated 96-well plate and allowed to attach for 24 h before starting the assay. A 7-point serial dilution of the drug was prepared in media at double the final concentration, and 100 μL of the treatment was added to each well in triplicate. Control treatments were cell media only and vehicle control, which was the drug vehicle (DMSO or water) at the same concentration as the highest drug concentration. The treated microtissues (or 2D cells) were incubated at 37 °C and 5% CO_2_ for 4 days. The CellTiter-Glo^®^ 3D assessed the effect of the drug on day 9 by measuring the ATP levels of the cells and was performed according to the manufacturer’s instructions, as outlined above in the viability assay section. The assay results were normalized to the average of the media control wells.

Additionally, assay results were compared to the reported maximum plasma concentrations (Cmax) from human pharmacokinetic studies. Reported concentrations were extracted from the literature [45,46,47,48,49] and converted to molar units (nM) using the molecular weight of each compound to enable direct comparison with in vitro dose–response data. These values were used to generate the red-dotted reference lines shown in the drug response plots.

### 2.10. Assessment of Hypoxia

The presence of hypoxia in microtissues was evaluated by HypoxiTRAK^TM^ (Biostatus, Shepshed, UK), a molecular probe that converts to a fluorescent metabolite in hypoxic cells and irreversibly accumulates. Microtissues for this assay were cultured from day 0 in phenol red-free media to prevent possible false-positive fluorescent signals of phenol red. Starting at day 5, the microtissues were cultured with 50 nM HypoxiTRAK^TM^ in culture media. Nuclei were stained with Hoechst 33342 (1:2000, Invitrogen, H3570) for 30 min. Hypoxia was assessed at days 7, 10, 14 and 21 by confocal microscopy (Zeiss LSM700).

### 2.11. Doxorubicin Diffusion Assay

Microtissue-cultured HCC1806 cells at day 5 were treated with 2.5 μM doxorubicin, and nuclei were stained with Hoechst 33342 (1:2000). After 30 min incubation in a humidified atmosphere at 37 °C and 5% CO_2_, live cell imaging was done for 12 h by confocal microscopy (Zeiss LSM700) with an image of the middle section of the microtissue taken every 30 min.

### 2.12. Statistical Analysis

Quantitative data were analyzed and visualized using GraphPad Prism 9 (v10.5.0). Drug treatment responses were analyzed using nonlinear regression (log[drug] versus response, three-parameter model). Flow cytometry data were analyzed using FlowJo (v10.10.0), and results are reported as the percentage of positive cells.

Differential gene expression analysis was performed in R (v4.4.2) using DESeq2 (v1.46.0). Raw count data were normalized and dispersion estimates calculated using default parameters, followed by a variance-stabilizing transformation for downstream analyses.

Principal component analysis (PCA) was performed on variance-stabilized data to assess global transcriptional variation among samples.

Differentially expressed genes were defined by a false discovery rate (FDR) < 0.05 and an absolute log2 fold change > 1. Gene ontology enrichment analysis was performed using clusterProfiler (v4.14.6), and results were visualized using enrichment maps (emapplots), where node size reflects gene count, node colour indicates adjusted *p*-value, and edge thickness represents term similarity.

## 3. Results

### 3.1. Cellular Architecture of the Microtissues

**Figure 2:**
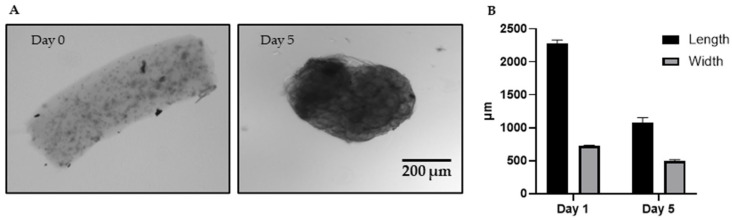
(**A**) Brightfield images of microtissue-cultured HCC1806 cells (2 × 10^6^ cells/mL of collagen) at day 0 and 5 (4× magnification, Scale bar is 200 µm). (**B**) Size assessment assay. The length (black) and width (grey) of microtissues were measured by the EVOS cell imager system. Each bar represents 45 microtissues (15 technical replicates per batch from three independent batches). Data are reported as mean ± SEM.

Microtissues were initially fabricated as cylindrical constructs measuring approximately 2 mm in length and 0.8 mm in diameter with minimal batch-to-batch variability (Figure 2). These dimensions were adapted from previously established modular tissue engineering protocols [36,44], in which similar sub-millimetre collagen modules have demonstrated sustained viability and mechanical stability. The cancer microtissues have a diameter that is 0.1 mm larger than the microtissues previously used for implantation. The selected geometry supports adequate diffusion, but with a slightly increased rate of nutrient gradient formation compared to the original diameter of the previous microtissues [50]. It also enables cell densities that support co-culture with other cell types while maintaining size compatibility for in vivo implantation studies (i.e., microtissues can pass through an 18G needle).

Following culture, progressive shrinkage of the microtissues was observed (Figure 2A). This contraction was attributed to cell-mediated remodelling of the surrounding collagen matrix, as proliferating cells engaged with the hydrogel and generated contractile forces. As the microtissues were free-floating and not constrained by the culture surface, cell-driven contraction resulted in an overall reduction in construct dimensions. In contrast, collagen constructs in contact with rigid culture surfaces experience mechanical constraint, which limits matrix contraction, as reported in other 3D culture systems [51]. The extent of shrinkage was cell type dependent, and construct size stabilized within the first five days of culture.

The selected cell density (2 × 10^6^ cells/mL) was optimized based on prior modular tissue engineering studies [36,44] as well as the known proliferation kinetics of cancer cells. This density represents a balance between achieving timely construct stabilization and preserving a biologically relevant progression from active proliferation to slower growth and possible quiescence/senescence, along with gradual emergence of hypoxia, mimicking key aspects of tumour evolution.

**Figure 3:**
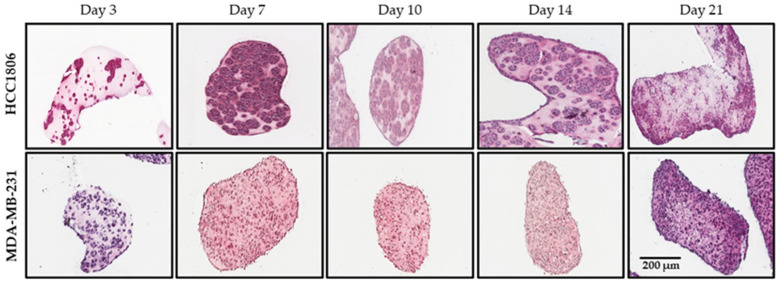
Morphology of microtissues. H&E staining of microtissue-cultured HCC1806 (**Top panel**) and MDA-MB-231 (**Bottom panel**) cells at day 3, 7, 10, 14 and 21 (4× magnification). As the HCC1806 microtissues age, the cells proliferate and grow in clusters. The clusters are highest in number at day 10, but cell clustering diminishes at later time points, and by day 21, most of the cells are found along the edges of the structure. Conversely, the MDA-MB-231 cells do not form large clusters and seem to spread uniformly all around the structure throughout the 21-day growth period. Images shown are representative of three independent experiments. Scale bar is 200 µm.

The histological examination of microtissue-cultured HCC1806 cells (Figure 3) over a 21-day period showed that by day 10, clusters of cells homogenously populated the collagen hydrogel and completely filled the structure. However, at later time points, cell clusters and their organized distribution were lost, and cells started to concentrate on the edges of the microtissue. Consequently, a hollow space started to reappear within the core, suggesting the migration of cells towards the edges of the structure. Similar results were seen with MDA-MB-231 cells; however, they typically formed only small clusters of cells compared to the HCC1806 (Figure 3). This hollowing out of the microtissues could indicate the establishment of an oxygen and nutrient gradient, and possibly a hypoxic core, which forces the cells to migrate toward the edges, similar to an actual tumour.

### 3.2. Gene Expression Profiles of Microtissue-Cultured TNBC Cells

**Figure 4:**
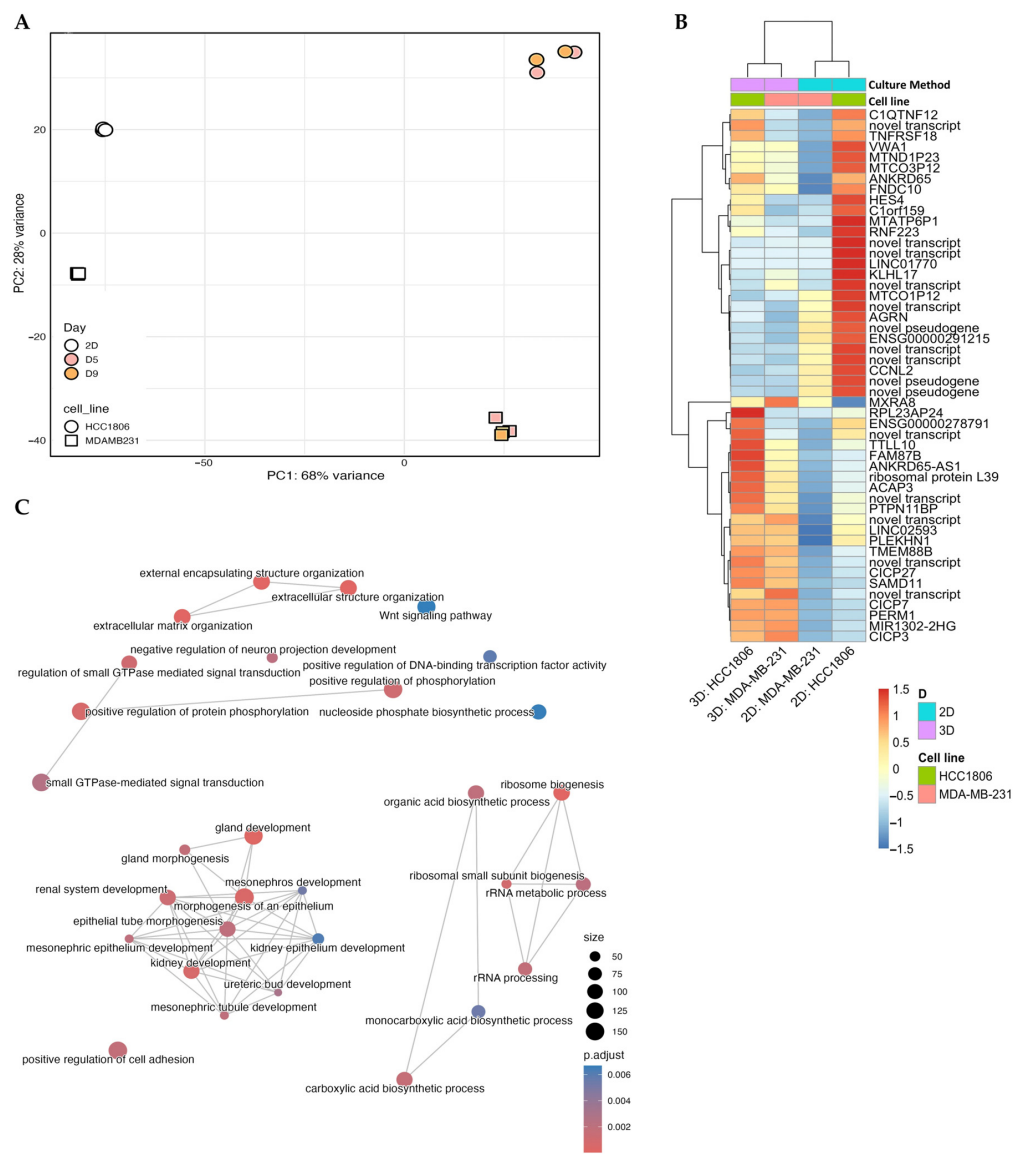
Transcriptomic and functional comparison of 2D-cultured versus 3D Microtissue- cultured breast cancer cell lines (HCC1806 and MDA-MB-231). (**A**) The Principal Component Analysis (PCA) plot displays the variance between samples. The x-axis (PC1) makes up 68% of the variance, and the y-axis (PC2) accounts for 28%. Data points are grouped by cell line (Circles: HCC1806, Squares: MDA-MB-231) and culture duration/type (2D and 3D Days 5 and 9), showing different clustering patterns based on cell type and culture duration/type. Each data point is an independent, biological replicate. (**B**) Hierarchical clustering heatmap shows differentially expressed genes and transcripts across two cell lines (HCC1806 and MDA-MB-231) in different culture conditions (2D and 3D). The top annotation bars indicate the culture dimension (Cyan: 2D, Purple: 3D) and cell line (Green: HCC1806, Salmon: MDA-MB-231). The colour scale represents relative gene expression (Z-score), ranging from blue (downregulated, −1.5) to red (upregulated, +1.5). (**C**) Network enrichment analysis shows the functional clustering of important gene ontology terms. Nodes represent biological processes (e.g., “extracellular matrix organization,” “ribosome biogenesis,” “Wnt signalling pathway”). The size of each node relates to the number of genes associated with the term (size count 50 to 150), and the node colour indicates statistical significance (*p*.adjust), with red indicating lower *p*-values (higher significance) and blue indicating higher *p*-values.

To validate the molecular impact of our model, we performed RNA sequencing on HCC1806 and MDA-MB-231 cells cultured in 2D monolayers versus microtissues (Figure 4, Tables S1–S4). Principal Component Analysis (PCA) revealed that the culture geometry was the primary driver of variance (PC1: 68%), confirming that the microtissue environment induces a profound transcriptomic shift compared to the 2D cell culture (Figure 4A). Furthermore, the two cell lines separated distinctively along PC2 (28%) within the 3D cluster, mirroring the heterogeneity observed in our functional assays. This global reprogramming was further characterized by hierarchical clustering (Figure 4B), which identified a robust ‘3D-specific’ gene signature shared by both cell lines, including the upregulation of matrix-associated genes such as MXRA8, AGRN and FNDC4. However, beyond this shared identity, pathway enrichment analysis (Figure 4C) revealed that the two cell lines engage distinct biological programmes. Notably, we observed the specific enrichment of ‘epithelial tube morphogenesis’ and ‘gland development’ pathways. This transcriptomic signature directly corroborates our histological analysis (H&E), which revealed that HCC1806 microtissues spontaneously organize into circular, milk duct-like clusters that were notably absent in the MDA-MB-231 microtissues. This confirms that the differential gene regulation observed in our model translates into distinct, cell-line-specific morphological phenotypes.

### 3.3. Microtissue-Cultured Cancer Cells Retain Their Viability and Proliferative Capacity

**Figure 5:**
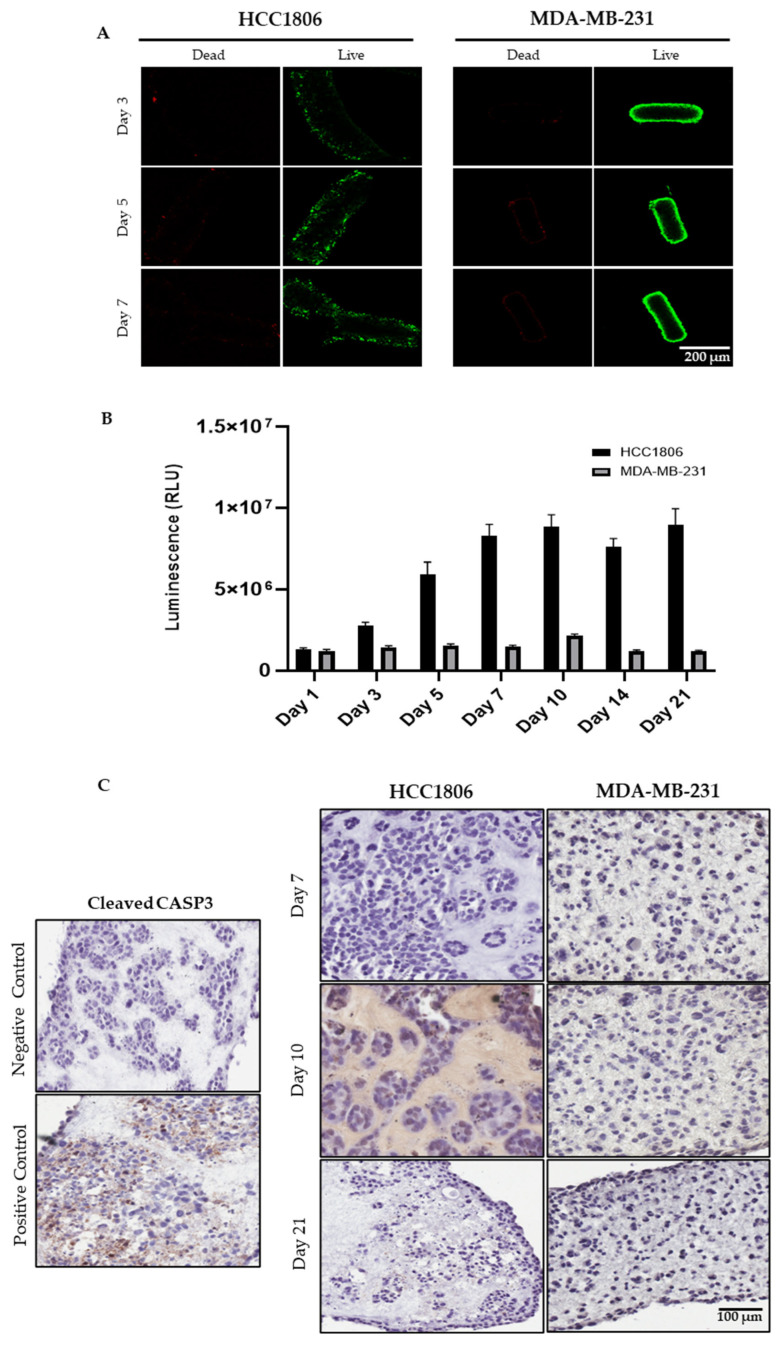
Viability and cell death in TNBC microtissues. (**A**) Representative images of live/dead staining of the 3D microtissue-cultured HCC1806 (**left panel**) and MDA-MB-231 (**right panel**) cells at days 3, 5, and 7, taken by confocal microscope. The shown images are representative of three independent experiments. The green staining is Calcein AM for live cells, and the red staining is SYTOX™ deep red stain for dead cells (5× magnification). Scale bar is 200 µm. (**B**) The viability of HCC1806 and MDA-MB-231 cells cultured in microtissues was measured based on ATP levels using CellTiter-Glo 3D during the 21-day growth period. Data are reported as mean ± SEM of three independent experiments. (**C**) Representative images of immunohistochemical staining of cleaved-Caspase 3 in HCC1806 and MDA-MB-231 microtissues at day 7, 10, and 21 (20×). The apoptotic cells are stained brown, and microtissue-cultured HCC1806 treated with the IC50 of doxorubicin were used as a positive control. An untreated microtissue is shown as a negative control. Images are representative results from three independent experiments. Scale bar is 100 µm.

It was essential to investigate the viability of cells in microtissues to demonstrate the capacity of our model in keeping cells alive. The viability of two different TNBC cell lines, HCC1806 and MDA-MB-231, was assessed over a 21-day period (Figure 5). A live/dead staining assay was initially performed to evaluate the viability of microtissue-cultured cells (Figure 5A). As seen with other cell types grown in modular tissue-engineered constructs [52,53], both tested cell lines maintained good cell viability after the formation of the microtissues. Additionally, this observation was confirmed by an ATP release assay to measure cell viability. As shown in Figure 5B, the HCC1806 cells proliferate over time as there is a continuous increase in ATP levels over the first 10 days and then the ATP levels plateau. In the case of MDA-MB-231, a certain level of ATP release was maintained over the 21-day growth period. Furthermore, cell death was assessed by the activated cleaved-Caspase 3 staining, and in both cell lines, a minimal number of apoptotic cells were observed in the microtissues (Figure 5C), confirming the live/dead staining. This suggests that the HCC1806 cells in the microtissues are most likely decreasing their proliferation rate, rather than undergoing apoptosis at later time points (day 10+) in the microtissues. In contrast, the microtissue-cultured MDA-MB-231 cells seem to remain stable throughout the 21-day growth period.

**Figure 6:**
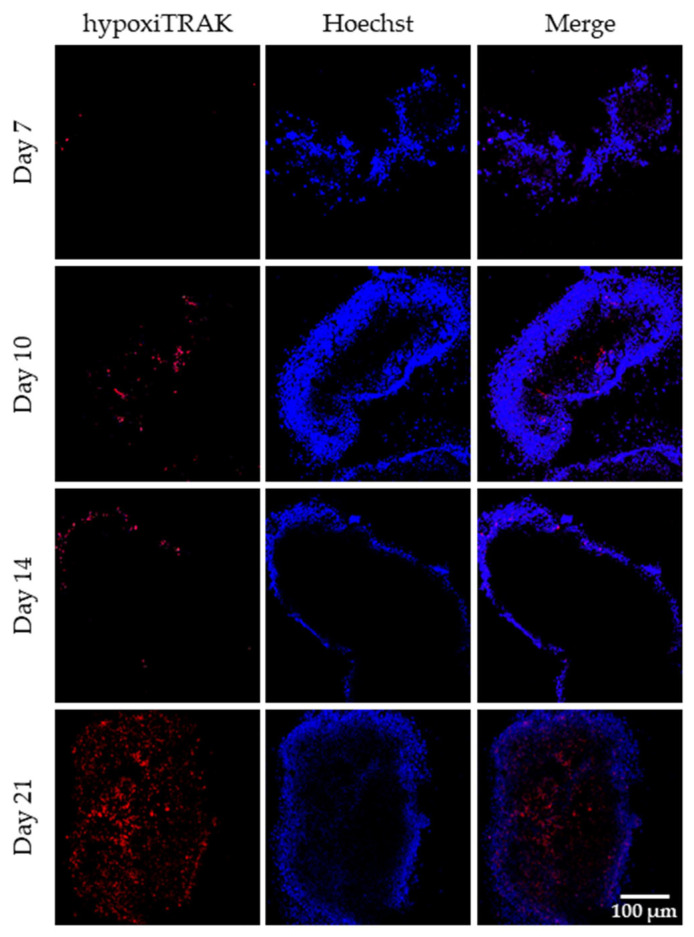
Evaluation of hypoxia. Hypoxia staining of HCC1806 microtissues at days 7, 10, 14, and 21 using HypoxiTRAK probe (red). Microtissues were incubated with 50 nM HypoxiTRAK from day 5 onward. Nuclei were counterstained with Hoechst 33342 (blue) for 30 min prior to imaging (10× magnification). Shown are representative images from three independent experiments. Scale bar is 100 µm.

We speculated that the observed decrease in cell proliferation within the microtissues could indicate the establishment of oxygen and nutrient gradients within the microtissue, and potentially a hypoxic core, which has also been seen in other hydrogel model systems [50]. The presence of hypoxia was evaluated by the hypoxiTRAK dye, which irreversibly accumulates in cells that have experienced a hypoxic event. As shown in Figure 6, several hypoxic cells were observed starting at day 10 and increased in number till day 21. Overall, it can be concluded that the microtissue architecture changes over time. At early time points (prior to day 10), there is optimal proliferation of the cancer cells within the microtissue, and as the cell density in the microtissue increases, the proliferation slows, and there is a hypoxic core formation within the microtissue.

### 3.4. TME-Driven Modulation of the CD44^+^/CD24^−^ Stemness Phenotype in TNBC Cells

**Figure 7:**
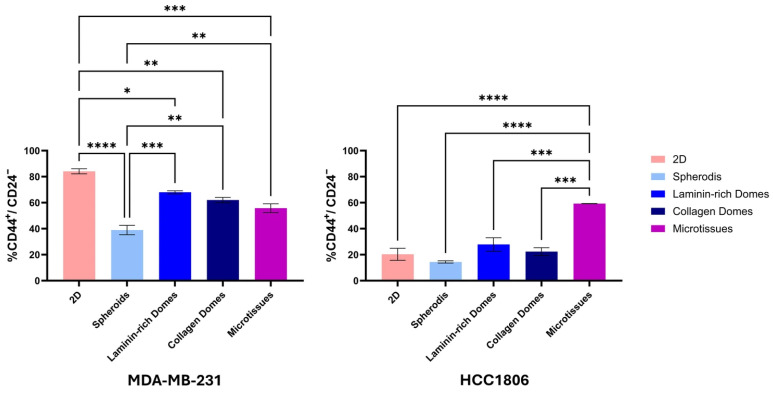
CD44^+^/CD24^−^ cancer stem cell populations across 2D and 3D culture systems. Quantification of the CD44^+^/CD24^−^ population in MDA-MB-231 (**left**) and HCC1806 (**right**) TNBC cell lines cultured in 2D monolayers, spheroids, collagen domes, laminin domes, and microtissues. Data are presented as mean ± SEM of three independent experiments. Statistical significance was determined using one-way ANOVA followed by Tukey’s multiple comparison test. * *p* < 0.05, ** *p* < 0.01, *** *p* < 0.001, **** *p* < 0.0001.

To benchmark the functional consequence of our model against established 3D systems, we quantified the CD44^+^/CD24^−^ cancer stem cell (CSC) population across microtissues, 2D monolayers, and standard 3D models (spheroids, collagen domes or laminin domes) (Figure 7). In the mesenchymal-like MDA-MB-231 line, all 3D models showed a reduction in the CSC phenotype compared to 2D controls; however, differences among the 3D systems, including microtissues, were not statistically significant except for the spheroid system, which has a major reduction in CD44^+^/CD24^−^ cell levels. This likely reflects a normalization of the cells’ phenotype, where the physiological stiffness of the 3D matrix mitigates the artificial mesenchymal activation typically induced by rigid 2D culture plastic [54,55]. In stark contrast, the epithelial-like HCC1806 line exhibited a dramatic enrichment of the CD44^+^/CD24^−^ population specifically in Microtissues (~60%), significantly surpassing the levels observed in 2D, spheroids, and dome cultures (*p* < 0.0001).

Although microtissues exhibited an increased proportion of CD44^+^/CD24^−^ cells, enrichment of this phenotype alone does not necessarily indicate expansion of functional stem-like populations. In primary breast tumours, CSC populations typically represent a minority fraction and are spatially regulated by microenvironmental cues. The enrichment observed in microtissues may reflect preservation of niche-dependent phenotypic plasticity rather than uniform expansion of a stem-like state. In contrast, 2D culture conditions can artificially alter cell phenotype through supraphysiological stiffness and loss of matrix signalling. Therefore, the relevance of the microtissue model lies in its capacity to support dynamic tumour-associated phenotypes within a defined extracellular matrix environment.

### 3.5. Cancer Microtissues as a Drug Screening Platform

An important aspect of cancer microtissue development is its utility as a drug screening platform. Microtissues containing either HCC1806 or MDA-MB-231 cells were screened for their response to chemotherapy drugs to evaluate their use as a drug screening platform. Drug dose–response curves were generated using a 7-point serial dilution ranging from 1 to 10,000 nM (0.001–10 µM). Based on the viability data (Figure 5), day 5 was chosen as the time point to start the drug assays. This is because the microtissues show a tissue architecture with the formation of multicellular groups of cells, and there is a large window of cell proliferation, and most cancer therapies target actively proliferating cells.

**Figure 8:**
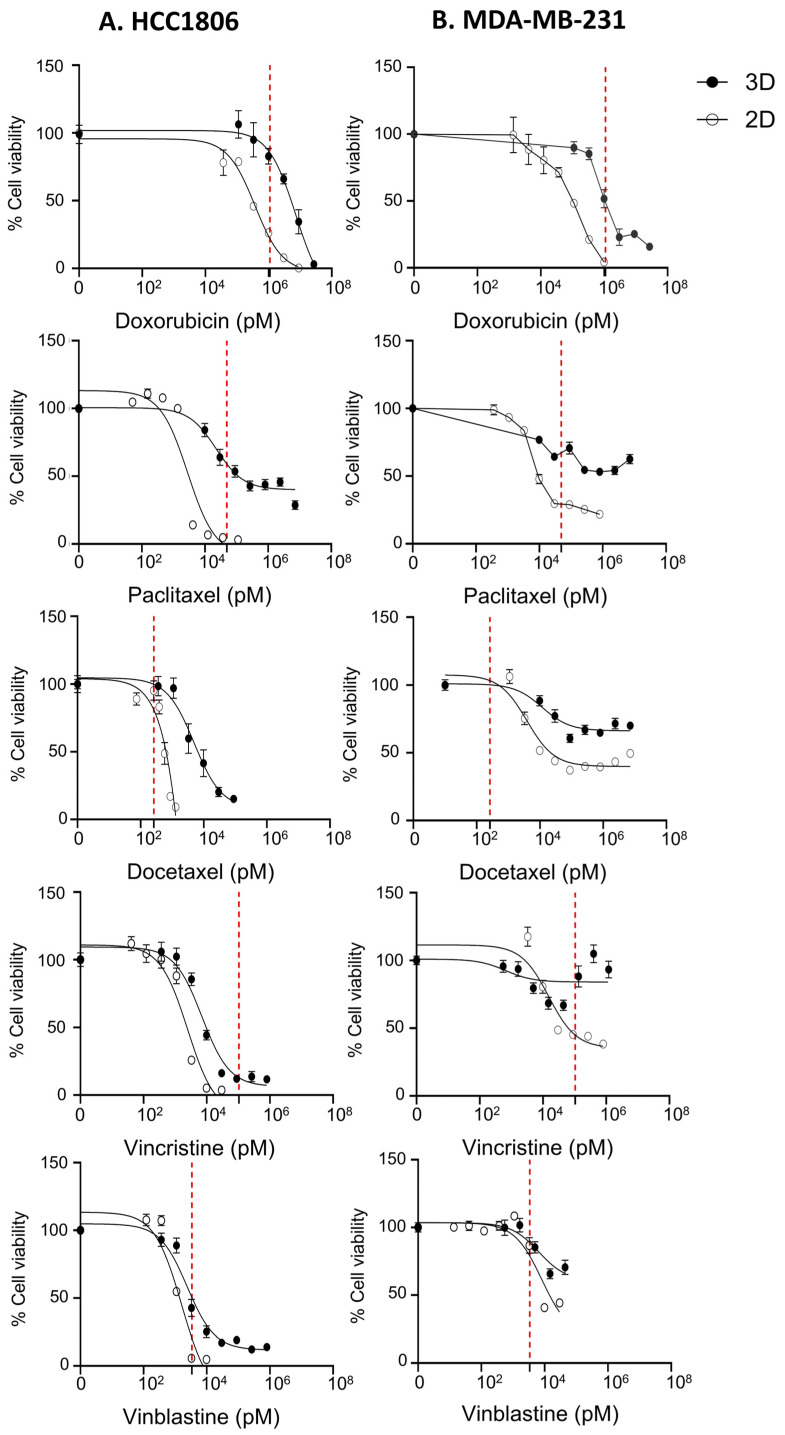
Dose–response curves of 2D-cultured (open circles) vs. 3D microtissue-cultured (closed circles) TNBC cells. HCC1806 (**A**) and MDA-MB-231 (**B**) cells were treated for 4 days with doxorubicin, paclitaxel, docetaxel, vincristine, or vinblastine. Cells were seeded at 1200 cells/well for 2D culture or 3 microtissues/well for 3D culture in 96-well plates. Each condition was assessed in triplicate wells (technical replicates). Data represent the mean ± SEM of three independent experiments. The red dotted line represents the maximum plasma concentration of each drug according to the literature for the given drugs [45,46,47,48,49].

The response of the HCC1806 microtissues was first tested with two common chemotherapy agents, paclitaxel and doxorubicin (Figure 8). According to the results, 2D-cultured cells were more susceptible to the tested drugs, compared to microtissue-cultured ones. Despite the fact that chemotherapeutics most often are able to completely eradicate 2D-cultured cancer cells in vitro, this does not correlate with their activity against actual patient tumours in the clinic [56]. As shown in Figure 8, either doxorubicin or paclitaxel treatment at the maximal plasma concentrations of the drugs [45,46,47,48,49] would not have been able to completely eradicate microtissue-cultured cells. The potency of all drugs was reduced in killing cells grown in microtissues. Additionally, efficacy was also diminished in all cases except the doxorubicin-treated HCC1806 microtissues. The decline of efficacy was the highest in the case of paclitaxel, where 50% or more of the cells survived paclitaxel concentrations that were 100× the IC50, indicating the emergence of a drug-resistant cell population.

**Figure 9:**
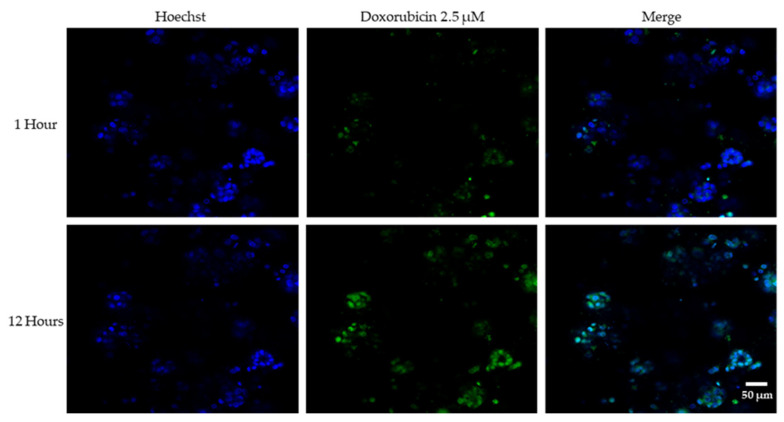
Doxorubicin diffusion assay. HCC1806 microtissues were treated with a 2.5 μM concentration of doxorubicin (Green), incubated with the nuclei stain Hoechst 33342 (Blue) for 1 h and imaged live for 12 h. Shown are representative images from three independent experiments at 1 h and 12 h (20×).

This resistance is not likely to be due to the diffusion limitations of the drug into the microtissue, as doxorubicin has completely penetrated the microtissues within 12 h (Figure 9 and Movie S1), and the drug treatment duration is 4 days. Therefore, the drug resistance is more likely due to changes in the cellular phenotype that resulted from the growth in the microtissue.

Paclitaxel is an anti-tubulin agent of the taxane class, which stabilizes the microtubules by blocking the disassembly of tubulin [57]. The specificity of the observed resistance to paclitaxel was tested with additional drug treatments. Starting with docetaxel, another taxane drug, but with higher affinity for tubulins compared to paclitaxel [58], it showed significantly higher potency in killing microtissue-cultured HCC1806 cells, as expected. Furthermore, a different class of anti-tubulin agents, the vinca alkaloids, which target polymerization of tubulins, was tested. As shown in Figure 8, neither vincristine nor vinblastine was able to eradicate all of the cells in microtissues, but the loss of efficacy of vinca alkaloids against microtissue-cultured cells was less prominent compared to paclitaxel treatment. In contrast to the basal cell line HCC1806, the mesenchymal MDA-MB-231 showed much higher resistance to all tested treatments (Figure 8). This was in accordance with the lower proliferation of the MDA-MB-231 cells and previous studies showing the dramatic difference between the sensitivity of these two cell lines to chemotherapy [59,60]. Overall, our data suggests the emergence of a paclitaxel-specific mechanism of resistance in microtissue-cultured HCC1806 cells.

## 4. Discussion

Preclinical studies play a central role in determining the success of novel therapeutics in human trials. Two-dimensional cell culture and animal models remain the primary tools used in early drug development. While 2D systems lack the architectural, mechanical, and microenvironmental complexity of human tumours, animal models remain essential for recapitulating tumour organization and systemic interactions, albeit at higher cost and lower throughput. Emerging 3D culture platforms aim to complement existing models by bridging the gap between traditional 2D culture systems and complex in vivo models.

Although numerous 3D tumour models [61,62,63,64,65] have been developed, many remain constrained by reproducibility challenges, matrix variability, or technical complexity. Laminin-rich matrices, such as Matrigel, exhibit batch-to-batch variability due to their undefined growth factor composition. Scaffold-free spheroids can display aggregation-dependent size heterogeneity. Microfluidic and tumour-on-chip systems offer dynamic control but often require specialized fabrication and handling. To address these limitations, we evaluated a collagen-based platform, called microtissues, derived from modular tissue engineering, as a biomimetic and practical in vitro tumour model.

Our data shows that microtissues rapidly remodel the ECM through cell-driven contractile forces, leading to structural stabilization within the first five days of culture. This behaviour aligns with observations in free-floating hydrogel systems, where the lack of attachment to rigid plastic enables natural tissue contraction [66]. Histological analysis (Figure 3) indicated that HCC1806 cells form organized duct-like clusters, while MDA-MB-231 cells remain more dispersed. These findings suggest that the microtissue environment preserves intrinsic cell line–specific traits that are often suppressed in 2D cultures.

Transcriptomic profiling (Figure 4, Tables S1–S4) further indicated that culture dimensionality was the dominant determinant of cellular state in this system. The transition from 2D to 3D culture accounted for the majority of gene expression changes, reflecting substantial reorganization of transcriptional programmes rather than minor modulation. The presence of a distinct 3D-associated gene signature, including upregulation of matrix-related genes such as MXRA8, AGRN, and FNDC4, supports enhanced ECM engagement and mechanochemical signalling in collagen-based microtissues compared with rigid plastic substrates [67,68,69].

Functional comparisons with spheroids and hydrogel domes showed that microtissues differentially influence cancer stem cell (CSC) characteristics (Figure 7). The significant increase in the CD44^+^/CD24^−^ population in HCC1806 microtissues (up to ~60%) exceeded that observed in other 3D models. However, increased stemness alone does not necessarily indicate expansion of functional CSCs. In primary breast tumours, CSC populations typically represent a minority fraction and are spatially regulated by microenvironmental cues [70]. The enrichment observed here may instead reflect preservation of niche-dependent phenotypic plasticity within a defined collagen matrix. In contrast, the reduction in CD44^+^/CD24^−^ markers in MDA-MB-231 within microtissues, relative to 2D culture, suggests partial normalization of mesenchymal traits that are enhanced under rigid plastic conditions. It is important to note that CD44^+^/CD24^−^ status is not universally applicable as a CSC marker across all breast cancer subtypes, particularly mesenchymal-like lines such as MDA-MB-231 [71]. Therefore, these findings should be interpreted as comparative phenotypic indicators rather than absolute quantification of stem cell frequency.

A key feature of solid tumours is the natural emergence of hypoxia. In many current in vitro cancer models, hypoxia is artificially induced using hypoxia chambers [72,73], resulting in uniform exposure of all cells to reduced oxygen levels. In contrast, our model demonstrates spatially emerging hypoxia (Figure 6) that arises as a consequence of proliferation-driven increases in cellular density, resulting in diffusion limitations within the construct.

Drug-response studies (Figure 8) revealed substantial differences between 2D and microtissue cultures. As expected, 2D-cultured cells demonstrated high sensitivity to treatment, whereas microtissue-cultured cells exhibited reduced responsiveness consistent with tumour-associated resistance phenotypes. Notably, resistance was observed even close to clinically relevant plasma concentrations [45,46,47,48,49]. This reduced responsiveness resulted in diminished potency of all tested drugs and, in some cases, such as paclitaxel, complete loss of efficacy. Even though taxanes and vinca alkaloids both target tubulins among tested drugs, resistance to paclitaxel was more prominent than all other tested drugs. This could stem from altered β-tubulin expression [74], increased drug efflux [75], unfolded protein response (UPR) [76], or microenvironment-driven phenotypic shifts. Future studies will focus on dissecting the molecular pathways underlying this selective resistance phenotype.

Doxorubicin diffusion analysis (Figure 9) confirmed complete drug penetration within 12 h, indicating that resistance was not attributable to limited drug delivery but rather to biological adaptations within the 3D microenvironment, including hypoxia and phenotypic plasticity. Although diffusion kinetics may differ among the other tested agents, the 4-day treatment duration likely permits their adequate penetration within the microtissues.

Treatment was initiated at day 5 following stabilization of construct size and establishment of microenvironmental features. We acknowledge that later time points, representing more equilibrated proliferative states, may further enrich slower-cycling or clonogenic populations and could yield additional insights into treatment resistance dynamics. Future studies will evaluate drug response across multiple maturation stages to better reflect tumour heterogeneity and therapeutic targeting of clonogenic subsets.

Despite the advantages demonstrated in this study, several limitations remain. The current model consists solely of cancer cells embedded within collagen and does not yet incorporate key stromal or immune components of the tumour microenvironment. Previous research using modular tissue engineering has shown its flexibility to characterize an optimal microenvironment for the growth and proliferation of various cell types, including cardiomyocytes [52], adipocytes [77], and pancreatic islet cells [53]. Additionally, the collagen hydrogel can be enriched with ECM components such as fibronectin and laminin [78], depending on the requirements of the tissue under study. Therefore, the complexity of this model can be further enhanced to better recapitulate additional features of the TME. This may involve incorporating non-cancerous cells, such as endothelial cells, fibroblasts, and macrophages, to recapitulate stromal and immune components that are absent in the current configuration.

Additionally, the system has not yet been validated using patient-derived tumour samples, nor has its predictive capacity been correlated directly with clinical outcomes. However, drug-response assays performed in this study demonstrated reproducible dose–response curves, with effective concentrations aligning with clinically relevant plasma levels. While these findings support the physiological relevance of the model, further work is required to evaluate its performance using patient-derived cells and to establish concordance between in vitro drug responses and patient treatment outcomes.

## 5. Conclusions

Our data demonstrates the capacity of modular tissue engineering to generate a biomimetic cancer model while maintaining the practical ease and cost-efficiency of 2D cell culture. These findings support the potential of microtissues as a useful and physiologically relevant in vitro platform for cancer research.

## Figures and Tables

**Table 1:** Comparative evaluation of commonly used 3D tumour culture systems. This table summarizes key operational and biological characteristics of spheroids, laminin-rich dome cultures (e.g., Geltrex/Matrigel), collagen domes, and microtissues. Parameters include fabrication yield, matrix remodelling capacity, required materials, approximate cost relative to each other, and batch-to-batch variability. Fabrication yield for domes and microtissues is calculated per mL of matrix volume. Spheroid yield is reported per standard 96-well plate format (one batch per well).

Features	Spheroids	Laminin-Rich Domes	Collagen Domes	Microtissues
**Fabrication yield**	96 constructs per 96-well plate (1 per well)	40 constructs per mL of Geltrex or Matrigel	40 constructs per mL of Collagen	160 constructs per mL of collagen
**Matrix Remodelling**	None (no exogenous matrix)	Limited (plastic-anchored)	Limited (plastic-anchored)	High (free-floating)
**Required Materials**	Ultra-low attachment plate	Geltrex or Matrigel	Purified Type I Collagen	Purified Type I Collagen Reusable Cutting plate
**Cost**	Moderate–Low	High	Low	Low
**Batch-to-batch variability**	No matrix variability; large size variability	High matrix variability; small size variability	Low matrix variability; small size variability	Low matrix variability; small size variability

## Data Availability

The raw data supporting the findings of this article can be made available by the corresponding author upon request. The CAD file for the 3D-printed cutting plate and, where feasible, printed cutting plates can also be made available upon request by contacting the corresponding author (dean.chamberlain@saskcancer.ca).

## Supplementary Materials

The following supporting information can be downloaded at: https://www.mdpi.com/article/10.3390/cancers18060935/s1, Movie S1: Doxorubicin diffusion assay movie; Supplementary Table S1: Top Differentially Expressed Genes in HCC1806: Microtissues Day 5 vs. 2D cell culture; Supplementary Table S2: Top Differentially Expressed Genes in HCC1806: Microtissues Day 9 vs. Day 5; Supplementary Table S3: Top Differentially Expressed Genes in MDA-MB-231: Microtissues Day 5 vs. 2D cell culture; Supplementary Table S4: Top Differentially Expressed Genes in MDA-MB-231: Microtissues Day 9 vs. Day 5.

## Abbreviations

The following abbreviations are used in this manuscript:

TME	Tumour microenvironment
ECM	Extracellular matrix
CSC	Cancer stem cell
TNBC	Triple negative breast cancer
