# Supplementary material for: A 3D Collagen-Based In Vitro Cancer Model Created Through Modular Tissue Engineering
Source: Cancers (Basel). 2026 Mar 13;18(6):935. doi: 10.3390/cancers18060935 (PMC13024935; doi:10.3390/cancers18060935)
Supplement: Supplementary file 1 [file cancers-18-00935-s001.zip › Supplementary Tables 1-4.docx]

**Table S1. Top Differentially Expressed Genes in HCC1806: Microtissues Day 5 vs 2D cell culture**. Differentially expressed genes identified by DESeq2 comparing 3D microtissue-cultured HCC1806 cells at Day 5 with 2D-cultured cells. Genes were filtered using FDR < 0.05 and |log₂ fold change| > 1. The table includes gene name, log₂ fold change, adjusted p-value (padj), and direction of regulation. Adjusted p-values reported as <1E−300 indicate values smaller than the minimum numerical precision displayed by the software and should be interpreted as extremely small but non-zero values.

| Gene name | log_2_FoldChange | p-value | Direction |
| --- | --- | --- | --- |
| MT-CO1 | -4.77669 | <1E-300 | Downregulated |
| ACTB | -2.98279 | 4.1E-264 | Downregulated |
| EEF2 | -3.90904 | 5.2E-246 | Downregulated |
| ACTG1 | -3.87062 | 6.2E-240 | Downregulated |
| EEF1A1 | -3.15736 | 1.4E-232 | Downregulated |
| IGFBP3 | -4.93885 | 5.9E-225 | Downregulated |
| RPS5 | -4.3326 | 2.2E-211 | Downregulated |
| RPS2 | -2.36672 | 6.2E-195 | Downregulated |
| TMSB4X | -5.1007 | 1.8E-189 | Downregulated |
| AGRN | -3.88179 | 1.3E-188 | Downregulated |
| MTCO1P12 | -4.27239 | 4.5E-184 | Downregulated |
| RPL6 | -3.61837 | 3.9E-160 | Downregulated |
| ITGB4 | -2.97839 | 8.5E-149 | Downregulated |
| B2M | -2.64146 | 3E-147 | Downregulated |
| RPL3 | -2.76536 | 2.6E-141 | Downregulated |
| KRT7 | -2.61964 | 2.1E-138 | Downregulated |
| PSAP | -3.276 | 1.7E-134 | Downregulated |
| MT-CO2 | -2.85743 | 8.8E-132 | Downregulated |
| RPL7A | -3.4482 | 8.6E-128 | Downregulated |
| JUP | -3.61265 | 2.1E-124 | Downregulated |
| RPL15 | -2.39164 | 1.5E-122 | Downregulated |
| ACTN4 | -4.38173 | 4.5E-119 | Downregulated |
| JUNB | -4.13126 | 3E-117 | Downregulated |
| CAV1 | -2.86724 | 2.9E-115 | Downregulated |
| RPL37 | -4.69509 | 8.3E-113 | Downregulated |
| FASN | -4.18837 | 1.9E-112 | Downregulated |
| RPLP1 | -3.72732 | 1.8E-107 | Downregulated |
| RPL8 | -3.89938 | 1.6E-104 | Downregulated |
| MYH9 | -2.20794 | 2.5E-103 | Downregulated |
| MT-ATP6 | -2.39062 | 8.2E-102 | Downregulated |
| EPS8L2 | -3.54914 | 2.5E-99 | Downregulated |
| TRIM28 | -2.69077 | 1.96E-96 | Downregulated |
| PDLIM1 | -2.95437 | 8.64E-96 | Downregulated |
| MT-ND1 | -2.96195 | 1.36E-95 | Downregulated |
| EZR | -1.59374 | 1.73E-95 | Downregulated |
| GLUL | -2.87885 | 3.11E-95 | Downregulated |
| RPL19 | -3.8457 | 3.58E-95 | Downregulated |
| PPIA | -2.69103 | 1.06E-93 | Downregulated |
| FLNB | -2.91441 | 2.71E-93 | Downregulated |
| FTL | -3.11635 | 2.99E-93 | Downregulated |
| CD46 | -2.99047 | 1.05E-91 | Downregulated |
| MT-RNR1 | -3.92905 | 2.18E-91 | Downregulated |
| GSTP1 | -3.22362 | 6.25E-90 | Downregulated |
| DDR1 | -2.87665 | 1.56E-89 | Downregulated |
| ANXA8 | -3.5497 | 6.61E-89 | Downregulated |
| PLXNB2 | -3.96521 | 9E-86 | Downregulated |
| TACSTD2 | -3.41634 | 9.23E-86 | Downregulated |
| RPL4 | -1.41563 | 3.58E-85 | Downregulated |
| PLEC | -1.76856 | 5.11E-85 | Downregulated |
| RACK1 | -2.72313 | 6.85E-85 | Downregulated |
| FBXL3 | 3.564947 | <1E-300 | Upregulated |
| PHLDA1-AS1 | 5.995333 | <1E-300 | Upregulated |
| ENSG00000257390 | 6.508995 | <1E-300 | Upregulated |
| ENSG00000257222 | 6.883996 | <1E-300 0 | Upregulated |
| ENSG00000257042 | 8.401786 | <1E-300 | Upregulated |
| ENSG00000256469 | 6.680406 | <1E-300 | Upregulated |
| ENSG00000256152 | 6.480135 | <1E-300 | Upregulated |
| ENSG00000256001 | 6.48588 | <1E-300 | Upregulated |
| PXN-AS1 | 6.951585 | <1E-300 | Upregulated |
| P2RX5-TAX1BP3 | 6.515034 | <1E-300 | Upregulated |
| ENSG00000255050 | 7.059583 | <1E-300 | Upregulated |
| KLC2-AS1 | 6.484884 | <1E-300 | Upregulated |
| KLC2-AS2 | 6.937223 | <1E-300 | Upregulated |
| ENSG00000254721 | 7.438633 | <1E-300 | Upregulated |
| ENSG00000254680 | 8.155152 | <1E-300 | Upregulated |
| ENSG00000254662 | 7.045688 | <1E-300 | Upregulated |
| UBQLN1-AS1 | 5.754267 | <1E-300 | Upregulated |
| HOXA-AS3 | 5.876524 | <1E-300 | Upregulated |
| UQCRB-AS1 | 5.078248 | <1E-300 | Upregulated |
| ANO1-AS1 | 7.223863 | <1E-300 | Upregulated |
| TUBA1B-AS1 | 7.268415 | <1E-300 | Upregulated |
| ENSG00000258034 | 5.874971 | <1E-300 | Upregulated |
| PPP1R12A-AS2 | 5.474475 | <1E-300 | Upregulated |
| ENSG00000261505 | 5.965019 | <1E-300 | Upregulated |
| ENSG00000261312 | 6.106549 | <1E-300 | Upregulated |
| LMO7-AS1 | 7.706428 | <1E-300 | Upregulated |
| ENSG00000260978 | 5.196284 | <1E-300 | Upregulated |
| ENSG00000260934 | 5.875233 | <1E-300 | Upregulated |
| ENSG00000260545 | 5.704389 | <1E-300 | Upregulated |
| CENPN-AS1 | 6.895548 | <1E-300 | Upregulated |
| ENSG00000260121 | 5.346242 | <1E-300 | Upregulated |
| ENSG00000260107 | 6.443187 | <1E-300 | Upregulated |
| ENSG00000259972 | 6.781742 | <1E-300 | Upregulated |
| ENSG00000259627 | 6.444234 | <1E-300 | Upregulated |
| ENSG00000259536 | 6.478558 | <1E-300 | Upregulated |
| TPM1-AS | 6.498928 | <1E-300 | Upregulated |
| ENSG00000259357 | 6.698747 | <1E-300 | Upregulated |
| ENSG00000258908 | 7.191418 | <1E-300 | Upregulated |
| ENSG00000258666 | 6.052406 | <1E-300 | Upregulated |
| ENSG00000258430 | 6.4313 | <1E-300 | Upregulated |
| ENSG00000258232 | 7.431238 | <1E-300 | Upregulated |
| ENSG00000258092 | 7.64014 | <1E-300 | Upregulated |
| IGHVII-44-2 | 6.623211 | <1E-300 | Upregulated |
| FAM83A-AS2 | 6.867964 | <1E-300 | Upregulated |
| ENSG00000253174 | 6.590371 | <1E-300 | Upregulated |
| GPAT4-AS1 | 6.608037 | <1E-300 | Upregulated |
| WWTR1-AS1 | 6.875578 | <1E-300 | Upregulated |
| NDUFB2-AS1 | 5.828732 | <1E-300 | Upregulated |
| LINC02067 | 5.519266 | <1E-300 | Upregulated |
| ENSG00000240401 | 7.134804 | <1E-300 | Upregulated |

**Table S2. Top Differentially Expressed Genes in HCC1806: Microtissues Day 9 vs Day 5.** Differentially expressed genes identified by DESeq2 comparing HCC1806 microtissues at Day 9 versus Day 5 to assess temporal transcriptional remodeling within the 3D environment. Genes were filtered using FDR < 0.05 and |log₂ fold change| > 1.

| Gene name | log_2_FoldChange | p-value | Direction |
| --- | --- | --- | --- |
| BRSK2 | -3.84646 | 4.33E-17 | Downregulated |
| ENSG00000279069 | -2.27398 | 1.41E-16 | Downregulated |
| TMEM255B | -4.29708 | 5.23E-16 | Downregulated |
| MFSD11 | -2.26433 | 5.46E-15 | Downregulated |
| ENSG00000286618 | -4.07279 | 7.99E-15 | Downregulated |
| TUBA1B-AS1 | -2.04631 | 3.1E-14 | Downregulated |
| ENSG00000226332 | -1.99707 | 3.43E-14 | Downregulated |
| ENSG00000263424 | -2.88884 | 5.11E-14 | Downregulated |
| PRKG1-AS1 | -2.39991 | 7.42E-14 | Downregulated |
| DHX9-AS1 | -2.64242 | 1.82E-13 | Downregulated |
| ANAPC11 | -2.10701 | 2.19E-13 | Downregulated |
| HAS2-AS1 | -2.35485 | 5.54E-13 | Downregulated |
| SLC7A11-AS1 | -2.40601 | 6.53E-13 | Downregulated |
| NBEA | -2.04152 | 1.46E-12 | Downregulated |
| ENSG00000253102 | -1.99495 | 1.58E-12 | Downregulated |
| ENSG00000273212 | -2.04443 | 1.71E-12 | Downregulated |
| ENSG00000274021 | -2.37571 | 1.9E-12 | Downregulated |
| ENSG00000258232 | -1.90517 | 2.25E-12 | Downregulated |
| ENSG00000204620 | -2.48097 | 4.09E-12 | Downregulated |
| MAGOH-DT | -2.65876 | 4.09E-12 | Downregulated |
| ENSG00000279827 | -1.91791 | 8.37E-12 | Downregulated |
| IKBKG | -2.31009 | 9.74E-12 | Downregulated |
| CCDC85B | -3.05153 | 1.21E-11 | Downregulated |
| ADCY3 | -2.24555 | 1.34E-11 | Downregulated |
| ENSG00000272540 | -1.76048 | 1.94E-11 | Downregulated |
| ENSG00000260304 | -2.07965 | 2.76E-11 | Downregulated |
| LAMA5 | -1.85509 | 2.87E-11 | Downregulated |
| ITFG2 | -1.87312 | 3.42E-11 | Downregulated |
| ENSG00000261266 | -2.08802 | 4.93E-11 | Downregulated |
| CIMIP2A | -1.77167 | 7.23E-11 | Downregulated |
| ENSG00000261602 | -1.94959 | 7.23E-11 | Downregulated |
| LCORL | -1.7895 | 7.89E-10 | Downregulated |
| INTS8 | -2.10276 | 9.7E-10 | Downregulated |
| ENSG00000228838 | -2.87658 | 2.55E-09 | Downregulated |
| ENSG00000223725 | -2.29418 | 3.79E-09 | Downregulated |
| FANCD2OS | -1.94835 | 5.18E-09 | Downregulated |
| PHYKPL | -2.04704 | 5.47E-09 | Downregulated |
| GPS2 | -1.58765 | 7E-09 | Downregulated |
| ENSG00000279762 | -1.89412 | 8.74E-09 | Downregulated |
| ENSG00000232335 | -2.52442 | 9.69E-09 | Downregulated |
| HYOU1-AS1 | -1.63373 | 1.34E-08 | Downregulated |
| ANO1-AS1 | -1.61187 | 2.3E-08 | Downregulated |
| ENSG00000241962 | -1.79254 | 3.38E-08 | Downregulated |
| ENSG00000231864 | -1.76034 | 3.41E-08 | Downregulated |
| KRR1 | -2.28802 | 3.58E-08 | Downregulated |
| ITGAE | -2.18796 | 3.7E-08 | Downregulated |
| CYP1B1-AS1 | -1.93786 | 5.02E-08 | Downregulated |
| FTSJ3 | -1.51859 | 5.25E-08 | Downregulated |
| SLA2 | -1.7144 | 6.46E-08 | Downregulated |
| FCHSD1 | -2.4393 | 8.05E-08 | Downregulated |
| EGILA | 3.622382 | 3.65E-25 | Upregulated |
| LMNTD2 | 3.733011 | 1.51E-23 | Upregulated |
| ENSG00000228417 | 3.987414 | 2.36E-19 | Upregulated |
| ARHGEF39 | 2.417052 | 2.79E-19 | Upregulated |
| NDRG1 | 3.666312 | 1.12E-15 | Upregulated |
| ENSG00000255446 | 4.798885 | 2.14E-15 | Upregulated |
| SYT15B | 2.863894 | 2.65E-15 | Upregulated |
| SLC9A3 | 2.631121 | 1.59E-13 | Upregulated |
| ADAMTSL4-AS1 | 2.988053 | 1.04E-12 | Upregulated |
| SYT15 | 2.744236 | 1.9E-12 | Upregulated |
| ACTA2-AS1 | 2.69235 | 1.97E-12 | Upregulated |
| ENSG00000289757 | 2.337303 | 2.25E-12 | Upregulated |
| TBPL1 | 3.407075 | 1.17E-11 | Upregulated |
| TXNIP | 4.086195 | 1.48E-11 | Upregulated |
| ENSG00000225864 | 2.169185 | 8.77E-11 | Upregulated |
| ANKRD13A | 2.525476 | 3.45E-10 | Upregulated |
| MYCL-AS1 | 2.961323 | 4.27E-10 | Upregulated |
| ICAM4-AS1 | 2.340063 | 7.89E-10 | Upregulated |
| ENSG00000263826 | 1.793292 | 1.01E-09 | Upregulated |
| BHLHE40-AS1 | 1.801917 | 1.82E-09 | Upregulated |
| SPMIP1 | 2.965915 | 3.79E-09 | Upregulated |
| MAK | 2.494251 | 8.55E-09 | Upregulated |
| KLF2P4 | 2.689655 | 2.34E-08 | Upregulated |
| ADAMTSL4-AS2 | 2.933135 | 5.17E-08 | Upregulated |
| CALCRL-AS1 | 2.604001 | 5.34E-08 | Upregulated |
| RASA4B | 2.909166 | 6.6E-08 | Upregulated |
| EIF4A2 | 1.854787 | 6.75E-08 | Upregulated |
| MIR205HG | 2.752613 | 8.53E-08 | Upregulated |
| ENSG00000230521 | 1.506128 | 1.58E-07 | Upregulated |
| ENSG00000258757 | 1.561957 | 1.75E-07 | Upregulated |
| MRPL43 | 2.194025 | 2E-07 | Upregulated |
| ENSG00000271581 | 1.550403 | 2E-07 | Upregulated |
| RASA4CP | 3.273827 | 2.32E-07 | Upregulated |
| NOP53-AS1 | 2.004309 | 2.32E-07 | Upregulated |
| LOXL2-AS1 | 1.697698 | 2.44E-07 | Upregulated |
| IGFBP3 | 2.27916 | 2.46E-07 | Upregulated |
| RPL13A | 1.754336 | 2.48E-07 | Upregulated |
| LDLRAD2 | 1.629919 | 2.48E-07 | Upregulated |
| KMO | 2.003125 | 4.53E-07 | Upregulated |
| ENSG00000259351 | 3.401641 | 5.23E-07 | Upregulated |
| ENSG00000290692 | 3.067975 | 6.62E-07 | Upregulated |
| G6PC3 | 1.916515 | 7.29E-07 | Upregulated |
| HPS3 | 4.897172 | 9.8E-07 | Upregulated |
| PDLIM1 | 2.065234 | 1.03E-06 | Upregulated |
| ENSG00000237768 | 1.430421 | 1.13E-06 | Upregulated |
| DTNB-AS1 | 2.693219 | 1.18E-06 | Upregulated |
| ENSG00000290678 | 3.120801 | 1.26E-06 | Upregulated |
| ENSG00000286636 | 2.216051 | 1.34E-06 | Upregulated |
| GAS5-AS1 | 1.584894 | 1.54E-06 | Upregulated |
| ENSG00000231563 | 3.520542 | 1.8E-06 | Upregulated |

**Table S3. Top Differentially Expressed Genes in MDA-MB-231: Microtissues Day 5 vs 2D cell culture.** Differentially expressed genes identified by DESeq2 comparing 3D microtissue-cultured MDA-MB-231 cells at Day 5 with 2D-cultured cells. Genes were filtered using FDR < 0.05 and |log₂ fold change| > 1. The table includes gene name, log₂ fold change, adjusted p-value (padj), and direction of regulation. Adjusted p-values reported as <1E−300 indicate values smaller than the minimum numerical precision displayed by the software and should be interpreted as extremely small but non-zero values.

| Gene name | log_2_FoldChange | p-value | Direction |
| --- | --- | --- | --- |
| VIM | -2.80335 | <1E-300 | Downregulated |
| PABPC1 | -3.61824 | <1E-300 | Downregulated |
| ACTB | -4.19409 | <1E-300 | Downregulated |
| FTL | -4.60354 | <1E-300 | Downregulated |
| GAPDH | -4.41876 | <1E-300 | Downregulated |
| EEF1A1 | -2.9142 | <1E-300 | Downregulated |
| ACTG1 | -4.324 | <1E-300 | Downregulated |
| MT-RNR2 | -2.45806 | <1E-300 | Downregulated |
| MT-RNR1 | -5.96658 | <1E-300 | Downregulated |
| AXL | -3.59081 | 9.2E-252 | Downregulated |
| ITGB1 | -2.0862 | 3.4E-232 | Downregulated |
| HSP90AB1 | -1.6029 | 5.1E-232 | Downregulated |
| HSPA8 | -2.64122 | 4.3E-227 | Downregulated |
| EEF2 | -4.08591 | 9.6E-225 | Downregulated |
| TMSB4X | -3.31043 | 4.6E-205 | Downregulated |
| EIF4G1 | -2.99154 | 6E-202 | Downregulated |
| PSMD2 | -3.0451 | 1.8E-199 | Downregulated |
| RACK1 | -3.16963 | 1.3E-195 | Downregulated |
| RPLP0 | -4.16507 | 3.2E-194 | Downregulated |
| TUBB4B | -3.80986 | 2.6E-190 | Downregulated |
| YWHAZ | -2.07753 | 7.4E-185 | Downregulated |
| KIF5B | -3.62576 | 1.5E-183 | Downregulated |
| EIF4G2 | -2.99784 | 5.9E-180 | Downregulated |
| YWHAE | -2.08282 | 7.9E-180 | Downregulated |
| FTH1 | -2.19729 | 1.3E-177 | Downregulated |
| RPL8 | -4.62825 | 5.9E-165 | Downregulated |
| TUBA1B | -3.2979 | 3.4E-162 | Downregulated |
| RPL6 | -4.17372 | 5.5E-161 | Downregulated |
| HNRNPA1 | -2.1496 | 9.6E-158 | Downregulated |
| CCN1 | -5.31924 | 1.7E-148 | Downregulated |
| ENO1 | -2.04798 | 6E-147 | Downregulated |
| PFN1 | -2.9297 | 2.5E-142 | Downregulated |
| RTN4 | -2.69098 | 1E-141 | Downregulated |
| TUBB | -2.92798 | 8.8E-138 | Downregulated |
| HNRNPH1 | -2.83026 | 9.4E-137 | Downregulated |
| RPL12 | -3.21194 | 2.8E-131 | Downregulated |
| RPS2 | -3.29992 | 9.6E-130 | Downregulated |
| ACTN4 | -2.38839 | 3.9E-129 | Downregulated |
| TOP2A | -2.41284 | 2.1E-128 | Downregulated |
| RPL3 | -2.42093 | 4.5E-127 | Downregulated |
| TMBIM6 | -3.11743 | 1.3E-126 | Downregulated |
| RPL4 | -2.54543 | 6.2E-126 | Downregulated |
| BZW1 | -3.40232 | 2.3E-125 | Downregulated |
| PPIA | -2.60406 | 2.1E-124 | Downregulated |
| TXNRD1 | -2.25031 | 4.1E-124 | Downregulated |
| TPM3 | -2.70815 | 1.2E-123 | Downregulated |
| QSOX1 | -2.41006 | 7.4E-123 | Downregulated |
| CCT5 | -2.32656 | 1.2E-119 | Downregulated |
| FASN | -3.73192 | 1.4E-119 | Downregulated |
| CAPN2 | -1.9403 | 2E-119 | Downregulated |
| SCYL3 | 3.694996 | <1E-300 | Upregulated |
| ENSG00000258666 | 5.243805 | <1E-300 | Upregulated |
| HIF1A-AS3 | 5.134039 | <1E-300 | Upregulated |
| PRC1-AS1 | 4.724911 | <1E-300 | Upregulated |
| AP1G2-AS1 | 6.538019 | <1E-300 | Upregulated |
| BAZ1A-AS1 | 5.007941 | <1E-300 | Upregulated |
| ENSG00000258745 | 4.482982 | <1E-300 | Upregulated |
| ENSG00000258749 | 5.404674 | <1E-300 | Upregulated |
| ENSG00000258757 | 4.984705 | <1E-300 | Upregulated |
| HIF1A-AS1 | 7.387221 | <1E-300 | Upregulated |
| RBM25-AS1 | 5.457607 | <1E-300 | Upregulated |
| ENSG00000258908 | 4.397255 | <1E-300 | Upregulated |
| ENSG00000259033 | 5.799698 | <1E-300 | Upregulated |
| ENSG00000259049 | 4.638964 | <1E-300 | Upregulated |
| ENSG00000259118 | 5.881985 | <1E-300 | Upregulated |
| GNRHR2P1 | 6.818711 | <1E-300 | Upregulated |
| ENSG00000259185 | 5.153129 | <1E-300 | Upregulated |
| ENSG00000259187 | 5.554802 | <1E-300 | Upregulated |
| ENSG00000259627 | 4.92814 | <1E-300 | Upregulated |
| ENSG00000259607 | 6.321483 | <1E-300 | Upregulated |
| ENSG00000259605 | 7.036327 | <1E-300 | Upregulated |
| CTDSPL2-DT | 5.524537 | <1E-300 | Upregulated |
| ALDH1A3-AS1 | 7.210415 | <1E-300 | Upregulated |
| ENSG00000259515 | 5.272316 | <1E-300 | Upregulated |
| ENSG00000258646 | 4.762987 | <1E-300 | Upregulated |
| TPM1-AS | 5.461939 | <1E-300 | Upregulated |
| ENSG00000259357 | 6.00875 | <1E-300 | Upregulated |
| ENSG00000259352 | 4.277469 | <1E-300 | Upregulated |
| ENSG00000259327 | 5.161843 | <1E-300 | Upregulated |
| USP3-AS1 | 4.533677 | <1E-300 | Upregulated |
| ENSG00000259238 | 6.296751 | <1E-300 | Upregulated |
| ENSG00000259201 | 4.652177 | <1E-300 | Upregulated |
| DUT-AS1 | 6.357605 | <1E-300 | Upregulated |
| ENSG00000259661 | 5.895617 | <1E-300 | Upregulated |
| ENSG00000258471 | 5.181017 | <1E-300 | Upregulated |
| ENSG00000258424 | 4.707594 | <1E-300 | Upregulated |
| ENSG00000256469 | 5.92495 | <1E-300 | Upregulated |
| ENSG00000256569 | 5.40467 | <1E-300 | Upregulated |
| ENSG00000256789 | 5.109656 | <1E-300 | Upregulated |
| ENSG00000256928 | 5.466513 | <1E-300 | Upregulated |
| ARHGEF17-AS1 | 5.577595 | <1E-300 | Upregulated |
| TMPO-AS1 | 5.22921 | <1E-300 | Upregulated |
| ENSG00000257181 | 4.330522 | <1E-300 | Upregulated |
| GATC | 5.028769 | <1E-300 | Upregulated |
| ENSG00000257270 | 4.735883 | <1E-300 | Upregulated |
| ENSG00000257286 | 6.008441 | <1E-300 | Upregulated |
| CNPY2-AS1 | 5.953399 | <1E-300 | Upregulated |
| TNS2-AS1 | 5.800789 | <1E-300 | Upregulated |
| ENSG00000257342 | 6.786676 | <1E-300 | Upregulated |
| ENSG00000257386 | 5.691642 | <1E-300 | Upregulated |

**Table S4. Top Differentially Expressed Genes in MDA-MB-231: Microtissues Day 5 vs Day 9.** Differentially expressed genes identified by DESeq2 comparing HCC1806 microtissues at Day 9 versus Day 5 to assess temporal transcriptional remodeling within the 3D environment. Genes were filtered using FDR < 0.05 and |log₂ fold change| > 1.

| Gene name | log_2_FoldChange | p-value | Direction |
| --- | --- | --- | --- |
| KRT7-AS | -1.32769 | 1.92E-15 | Downregulated |
| ENSG00000261602 | -1.25687 | 5.63E-13 | Downregulated |
| ENSG00000272078 | -1.88516 | 2.02E-07 | Downregulated |
| PDE7B-AS1 | -1.33898 | 6.45E-07 | Downregulated |
| ENSG00000285520 | -1.92706 | 1.26E-05 | Downregulated |
| ENSG00000287051 | -1.88526 | 2.95E-05 | Downregulated |
| ENSG00000270823 | -1.27074 | 0.000356 | Downregulated |
| RELL1 | -1.22557 | 0.000422 | Downregulated |
| PDXP-DT | -1.38179 | 0.000501 | Downregulated |
| ENSG00000267774 | -1.69365 | 0.002197 | Downregulated |
| PPP1R12B | -1.67454 | 0.005449 | Downregulated |
| LINC01356 | -1.12865 | 0.024481 | Downregulated |
| ENSG00000260466 | -1.53115 | 0.030732 | Downregulated |
| KRT7 | -1.94621 | 0.035117 | Downregulated |
| FBXO44 | -1.44192 | 0.049415 | Downregulated |
| ENSG00000272114 | 1.761408 | 3.35E-29 | Upregulated |
| TAF9B | 1.690593 | 8.26E-20 | Upregulated |
| CST4 | 1.959609 | 1.4E-17 | Upregulated |
| NDRG1 | 2.388133 | 8.54E-11 | Upregulated |
| ENSG00000237768 | 1.126995 | 5.91E-09 | Upregulated |
| MT-ND1 | 1.121553 | 4.04E-06 | Upregulated |
| ENSG00000228417 | 1.992162 | 5.44E-06 | Upregulated |
| ENSG00000280026 | 1.214909 | 1.01E-05 | Upregulated |
| TCAF2 | 2.105542 | 1.75E-05 | Upregulated |
| WFDC13 | 1.766955 | 2.05E-05 | Upregulated |
| ENSG00000257764 | 1.169126 | 2.31E-05 | Upregulated |
| ENSG00000286670 | 1.161114 | 2.47E-05 | Upregulated |
| PCDHB1-AS1 | 1.23808 | 2.95E-05 | Upregulated |
| MTERF4 | 1.021877 | 3.29E-05 | Upregulated |
| TCAF2C | 2.042527 | 3.79E-05 | Upregulated |
| PPIL2 | 1.565044 | 4.06E-05 | Upregulated |
| C4B | 1.807581 | 6.21E-05 | Upregulated |
| CST1 | 2.326243 | 8.66E-05 | Upregulated |
| NR2F1-AS1 | 1.293369 | 8.66E-05 | Upregulated |
| EGILA | 1.111991 | 8.66E-05 | Upregulated |
| HPS3 | 2.183215 | 0.000214 | Upregulated |
| C4A | 1.731754 | 0.000385 | Upregulated |
| BEND3 | 1.463031 | 0.001081 | Upregulated |
| ZNF436-AS1 | 1.247064 | 0.00158 | Upregulated |
| ENSG00000291149 | 1.247996 | 0.001911 | Upregulated |
| MYBBP1A | 1.19193 | 0.002771 | Upregulated |
| VEGFA | 1.714908 | 0.003563 | Upregulated |
| CDCA7L | 1.317221 | 0.007377 | Upregulated |
| MUC5B-AS1 | 1.450123 | 0.007926 | Upregulated |
| CST2 | 1.362312 | 0.008166 | Upregulated |
| TGFBI | 1.346676 | 0.008266 | Upregulated |
| A2M-AS1 | 1.682507 | 0.013647 | Upregulated |
| IGFBP3 | 1.665231 | 0.015558 | Upregulated |
| OR2I1P | 1.663435 | 0.016091 | Upregulated |
| SRFBP1 | 1.623557 | 0.017391 | Upregulated |
| ENSG00000262884 | 2.216781 | 0.017993 | Upregulated |
| PGK1 | 1.10402 | 0.019744 | Upregulated |
| MGST2 | 1.610675 | 0.025638 | Upregulated |
| ENSG00000250619 | 1.739084 | 0.025638 | Upregulated |
| XPO5 | 1.179199 | 0.026821 | Upregulated |
| STC1 | 1.69459 | 0.026821 | Upregulated |
| ENSG00000258168 | 1.855317 | 0.026821 | Upregulated |
| ENSG00000226329 | 1.433204 | 0.045047 | Upregulated |
