# Supplementary material for: A 3D Collagen-Based In Vitro Cancer Model Created Through Modular Tissue Engineering
Source: Cancers (Basel). 2026 Mar 13;18(6):935. doi: 10.3390/cancers18060935 (PMC13024935; doi:10.3390/cancers18060935)
Supplement: Supplementary file 1 [file cancers-18-00935-s001.zip › Supplementary Figure. Movie 1/Supplementary Fig Movie 1.pdf]

Movie 1. Doxorubicin diffusion assay movie. HCC1806 microtissues were treated with 2.5 μM concentration of doxorubicin (Green), incubated with the nuclei stain Hoechst 33,343 (Blue) for 1 hour and imaged live every 30 min for 12 hours. Time-lapse movie over 12 hours showing doxorubicin diffusion through the HCC1806 microtissues. (20X)
